# Effects of electricity outages on enterprise productivity in Egypt: Lessons learned

**DOI:** 10.1371/journal.pone.0329479

**Published:** 2025-09-16

**Authors:** Hassan Aly, Fatma Ahmed

**Affiliations:** 1 Nile University, Cairo, Egypt; 2 Ohio State University, Columbus, Ohio, United States of America; 3 Formally, University of Saskatchewan, Saskatoon, Canada; Roma Tre University: Universita degli Studi Roma Tre, ITALY

## Abstract

This study investigates the impact of advanced electricity outage announcements on the operational efficiency of small and medium enterprises (SMEs), in Egypt, using profitability as a key performance indicator. Leveraging data from “Transition to Clean Energy Enterprise Survey” and applying the inverse probability-weighted regression adjustment (IPWRA) method to address selection bias, we estimate how outage predictability influences firm outcomes. We find that SMEs receiving advance notice of power disruptions are significantly more likely to achieve higher profitability compared to those without such information. The benefits are most evident among larger firms and sectors such as transportation, financial services, and accommodation, where operational planning is critical. While the policy partially offsets losses from outages, firms in areas with frequent blackouts still face substantial profitability challenges, highlighting the limits of transparency alone. Our findings emphasize that advance announcements enhance SME resilience by enabling adaptive measures, but long-term solutions require complementary infrastructure investments in high-risk regions. The study advocates for policy frameworks centered on transparency and rational expectations, demonstrating how proactive communication in public services can bolster economic resilience amid global uncertainties. These insights are particularly relevant for developing economies seeking to balance immediate crisis management with sustainable energy infrastructure development.

## Introduction

Insufficient infrastructure and inadequate electricity service quality remain persistent barriers to economic productivity and business performance across many developing countries and remote communities [1–4]. These constraints not only increase production costs but also force firms to rely on less efficient technologies, thereby reducing their competitiveness both domestically and internationally [5,6]. In fact, previous studies consistently emphasize the pivotal role that reliable electricity infrastructure plays in supporting economic growth and advancing development outcomes [5,7–19]. Nevertheless, despite this well-documented relationship, many developing economies continue to face unreliable electricity access. While firms may be formally connected to the grid, frequent scheduled and unscheduled outages severely disrupt operations [10]. This unreliability stems largely from the persistent gap between growing electricity demand and the system’s limited supply capacity [20]. Specifically in Egypt, electricity shortages present a significant obstacle to the productivity of small and medium-sized enterprises (SMEs). SMEs account for nearly 75% of private sector employment and contribute substantially to GDP, particularly in sectors such as manufacturing, retail, and services. However, repeated outages, especially in underserved rural areas, have become commonplace, with scheduled disruptions often lasting up to three hours per day. In fact, certain regions like Alexandria governorate experience even longer outages, whereas affluent areas such as Sheikh Zayed remain mostly unaffected. According to the ministry of electricity, these disparities are not primarily due to insufficient generation capacity but are instead driven by fuel shortages, particularly of natural gas and fuel oil [21].

The economic consequences of these outages are far-reaching. On one hand, many firms must divert resources to invest in costly diesel generators, which limits their ability to fund innovation and growth [16]. On the other hand, firms without access to backup power face complete production halts, leading to idle labor, spoilage of goods, and potential equipment damage [15]. As a result, businesses not only face immediate operational losses but also long-term competitive disadvantages due to higher input costs and disrupted supply chains. Furthermore, the lack of affordable alternative energy sources exacerbates the challenge for firms operating with thin profit margins. To mitigate these disruptions, the Egyptian government has introduced several reforms. Notably, since August 2022, a policy requiring advance notice of planned outages was implemented. This initiative aims to bring predictability to an otherwise uns1` energy environment and allows firms to prepare more effectively. Historically, Egypt's electricity supply has lagged behind rapid population growth and industrial expansion. Particularly in the 2000s and early 2010s, the country faced frequent load-shedding during periods of peak demand to avoid grid collapse [9,14]. The summer season, characterized by high energy consumption, continues to strain the grid and elevate blackout risks [21,22].

In response, Egypt launched major infrastructure projects. For example, the 2014 collaboration with Siemens resulted in three large-scale power plants, which added 14.4 gigawatts to the national grid, enough to supply energy to 40 million households [14,23]. Additionally, Vision 2030 outlines the country’s commitment to renewable energy integration and long-term energy security. Yet, grid instability and regional disparities persist, particularly in areas with aging infrastructure and accelerating demand [12,18]. For SMEs, the stakes are especially high. Due to limited financial flexibility, these firms are often unable to invest in alternative energy solutions. As a result, unanticipated outages lead to production delays, revenue losses, and even reputational harm [13,24]. Moreover, the unpredictability of power cuts impedes planning, making it difficult for businesses to manage inventories, coordinate staffing, or reschedule workflows. With that being said, the introduction of the advance notification policy marks an important step. While it does not eliminate outages, it helps businesses adjust production schedules, reassign tasks, or temporarily relocate operations. In doing so, it acts as a buffer that reduces the economic impact of service interruptions [25,26].

Hence, this paper makes a twofold contribution to the literature. First, it evaluates the effectiveness of Egypt’s advance outage notification policy in supporting SME productivity. Drawing on data from the economic research forum’s transition to clean energy enterprise survey and applying the inverse probability-weighted regression adjustment (IPWRA) method, the study investigates whether firms receiving advance notifications perform better in terms of profitability. While prior studies have explored the general productivity impacts of electricity shortages [5,8,9], few have examined whether enhanced predictability serves as an effective adaptation mechanism. Ultimately, this analysis contributes to broader conversations around energy policy, business resilience, and adaptive strategies in resource-constrained settings. The case of Egypt offers critical lessons for other emerging economies seeking to improve energy reliability while maintaining economic competitiveness.

The remainder of this paper proceeds as follows: we begin by reviewing the relevant literature, followed by an outline of the empirical methodology. We then present and discuss the findings and conclude with key policy implications and directions for future research.

## Literature review

In many developing countries, electricity provision remains largely under public control. Chronic underinvestment and outdated infrastructure continue to undermine service reliability. At the same time, private sector engagement in energy supply remains limited, constrained by regulatory barriers and investor preference for safer, more profitable ventures [27,28]. Egypt illustrates these dynamics well. In recent years, persistent energy shortages and budgetary constraints have led to repeated electricity cutbacks, which have raised serious concerns regarding both economic competitiveness and basic human rights [14,21]. These recurring outages highlight the urgent need to understand how unreliable electricity affects firms.

A growing body of empirical research examines how power interruptions shape production efficiency, input costs, technology choices, and overall business resilience [5,16,29]. For example, Abdisa [7] finds that frequent outages in Ethiopia reduce manufacturing productivity by 15–20%, mainly due to idle labor and equipment damage. In a broader cross-country study, Apeti and Ly [10] estimate that a 10% increase in outage frequency lowers total factor productivity (TFP) by 3.2% in low-income countries. Most studies rely on firm-level panel uses OLS and Tobit models to report statistically significant negative relationships between power outages and firm performance, controlling for firm size, sector, and asset ownership such as generators. Nevertheless, these standard models often struggle with endogeneity, particularly when infrastructure quality correlates with unobserved firm or location characteristics. To address this, Cole et al., [29] use an instrumental variable strategy, employing climate-induced variation in hydropower availability to isolate the exogenous component of electricity reliability. Their results provide credible causal estimates of outage effects on firm sales. Further, Fisher-Vanden et al., [16] use panel data from China to explore how firms respond to electricity shortages. Their study finds that firms adapt through costly strategies such as outsourcing and input substitution, thereby increasing unit production costs. Additionally, Cissokho [30] combines nonparametric data envelopment analysis (DEA) with regression models to examine how outage duration influences technical efficiency. Though rarely applied in African settings, the study discusses how electricity quality shapes productivity. Additionally, Adanlawo et al., [31] provide further evidence by reporting average outage frequencies and generator usage patterns in power-deficient regions.

Notably, only a few studies adopt formal causal inference frameworks. Chowdhury et al., [20] apply Propensity Score Matching (PSM) to measure how electricity access affects SME labor productivity in Bangladesh. Similarly, Kamanyire et al., [32] use both PSM and inverse probability weighting with regression adjustment (IPWRA) to estimate the impact of electrification on rural business formation in Uganda. Abeberese et al., [5] use a natural experiment, exploiting Ghana’s power rationing crisis to estimate causal effects of outages on firm output. They report that firms facing daily outages suffer output losses of 8–12%, with small enterprises hit hardest due to their limited backup capacity.

With that being said, our study applies the IPWRA approach to tackle both selection bias and unobserved heterogeneity in the Egyptian context. The method’s double robustness ensures consistent estimates as long as either the treatment or outcome model is correctly specified, thereby enhancing the credibility of causal claims even under imperfect specifications. Although Egypt-specific studies remain limited, relevant patterns emerge. Mohamed [14] highlights how localized load-shedding disrupts industrial zones, causing delays and heavy reliance on costly generators, which in turn erode profit margins. Chen et al., [12] provide supporting global evidence by linking extended outages to GDP contractions, raising concern for energy-intensive export sectors like textiles and chemicals in Egypt. Needless to say, how firms experience and respond to outages varies significantly by size, sector, and informality. Nzepang et al., [17] show that informal firms in Cameroon, despite lacking grid access, often perform better during outages due to flexible processes and decentralized energy use. In contrast, Hardy and McCasland [13] find that small formal firms in Ghana often reduce investments or downsize when faced with frequent outages, while larger firms absorb shocks through generators or relocating operations. Unsurprisingly, Egypt’s strained grid and recent subsidy reforms have worsened the situation. SMEs, which constitute about 75% of Egypt’s private sector, face existential threats from unplanned outages as most cannot afford diesel generators or solar alternatives [23]. Indeed, heavy industries such as steel and cement, which are vital to public infrastructure development, also incur significant operational costs, potentially discouraging foreign investment [33].

At the macro level, outages hamper growth by disrupting supply chains and inflating costs. Andersen and Dalgaard [9] estimate that power shortages reduce GDP growth by 1–2% annually in sub-Saharan Africa, with climate-driven risks worsening the burden [34]. A review by Avordeh et al., [35] confirms consistent negative effects of outages on SMEs, especially in low-income regions. Alby et al., [1] also highlight how firms in power-scarce environments resort to costly self-generation, often seen as inefficient second-best solutions. Fried and Lagakos [6] use a dynamic general equilibrium model to show that even small, short-run outages lead to significant long-run welfare losses through reduced firm entry, distorted resource allocation, and lower growth. Given Egypt’s projected 6% annual increase in electricity demand [14], such outages seriously threaten the country’s Vision 2030 development goals.

Yet, policy responses have mostly focused on short-term relief. Egypt’s load-shedding remains centrally managed, often with regressive effects that hit low-income areas hardest [21]. While De Nooij et al., [36] propose rationing schemes that minimize social loss, Egypt’s implementation still falls short. In contrast, countries like Ghana and India have introduced targeted subsidies and demand-response incentives to enhance resilience and supply-demand alignment [5,37]. Furthermore, recent literature emphasizes the value of integrating renewable energy to ensure both reliability and sustainability [38,39]. In particular, Egypt’s high solar potential and projects like the Benban Solar Park can serve as strategic assets, especially when paired with decentralized microgrids [14,23,33]. Nevertheless, the energy transition remains hindered by financial and regulatory barriers. Kim [40] suggests that faster clean energy adoption in emerging economies depends on simplified permitting and tailored fiscal incentives, especially for SMEs.

Another critical but under explored issue is the role of advance outage announcements. Timely information allows firms to reorganize operations, reduce downtime, and deploy backups effectively. For example, Woo et al., [41] find that early warnings in Hong Kong helped households and small firms shift power-intensive tasks, limiting economic losses. Similarly, De Nooij et al., [36] show that optimal rationing with transparent communication minimizes disruption costs. In contrast, unplanned cuts increase damage: Carlsson et al., [11] report that unexpected outages in Ethiopia raised manufacturing costs by up to 30%. Baarsma and Hop [25] find that consumers are willing to pay more for accurate outage forecasts, which help reduce uncertainty. Furthermore, Martin and Rice [42] show that storage systems combined with advance warnings improve grid resilience during climate events. Yum et al., [43] provide similar evidence from Hurricane Irma, while Salman et al., [44] point out the importance of early warning systems in reducing both outage duration and severity. Hence, this study aims to estimate the causal impact of electricity outages on firm productivity in Egypt, with a particular focus on SMEs operating in energy-intensive sectors. By leveraging the IPWRA method, the research contributes to the broader literature on infrastructure and development economics by providing robust, context-specific evidence from a key MENA economy. Moreover, it offers practical insights for policymakers seeking to enhance grid resilience, improve outage communication, and promote equitable access to reliable energy services.

## Model and data

To examine the effect of the power outage advance announcement on firm revenue we begin our analysis with OLS and probit regression. Then we exploit the augmented inverse probability weighting regression adjustment (IPWRA) estimator method designed to adjust for differences in observable characteristics between treated and untreated groups. The IPWRA estimator, often referred to as Wooldridge’s [45] “double-robust” method, integrates two techniques: regression adjustment (RA) and inverse probability weighting (IPW). The RA component estimates treatment effects by modeling expected outcomes based on observed covariates, while IPW accounts for the probability of treatment assignment. Together, they produce robust estimates of causal effects. In practical terms, this approach generates two predicted outcomes for each firm: one reflecting the scenario in which the firm received an advance outage announcement, and another representing the scenario in which the firm did not. This allows for a comprehensive comparison of the potential effects of the policy intervention across all firms in the sample. To put it simply, if the model used to predict treatment assignment (such as a logistic regression for the propensity score) is not fully accurate but the model predicting outcomes is valid, the estimator can still produce reliable estimates. Conversely, if the outcome model is misspecified but the treatment model is correctly estimated, the estimator will still work. This dual pathway to consistency provides a form of insurance against model misspecification [46]. This robustness makes IPWRA particularly attractive for causal inference. In contrast, other estimators typically require correct specification of their sole underlying model to ensure unbiasedness. Thus, the double robustness of IPWRA enhances credibility in empirical research by reducing dependence on the correctness of any single model.

To investigate the effect of the advanced announcement policy for electricity outages on firms’ efficiency $Y_{i}$, we assumed that a function of ($D_{i}$), a vector of explanatory variables ($X_{i}$), and the error term $\varepsilon_{i\text{\textbackslash{} }}$ expresses the efficiency.


$$Y_{i}=\;f(D_{i}\text{,}X_{i})\;+\varepsilon_{i}$$ (1)

Since the regions impacted by electricity outages and the associated policies for notification are determined by government action rather than random processes, using statistical methods like ordinary least squares (OLS) or Probit could lead to selection bias in the results. To address the possibility of this bias, this study opts for the regression adjusted inverse probability weighted estimator (IPWRA) instead of the propensity score model (PSM) [47,48]. Using IPWRA allows for more reliable estimates, accurately reflecting the effects of policies on relevant outcomes.

To estimate the effect of the electricity outage advance announcement policy on firm performance ($Y_{i}$), we use the inverse probability weighted regression adjustment (IPWRA) method. This approach combines propensity score weighting and regression adjustment to obtain doubly robust estimates of the average treatment effect on the treated (ATET).

First, we estimate the propensity score, the conditional probability of receiving the treatment given covariates using a Probit model:


$$f\left({\text{X}}_{\text{i}}\right)=P\left(D_{\text{i}}=\text{1}|{\text{X}}_{\text{i}}\text{\textbackslash{} }\right)$$ (2)

Where $f\left({\text{X}}_{\text{i}}\right)$ is the propensity score, the conditional probability that firm i received the treatment (advance outage announcement) given its observed characteristics. $D_{\text{i}}\text{\textbackslash{} }$is a binary treatment indicator, equal to 1 if firm i received the advance announcement, and 0 otherwise.

Propensity weights are constructed using simple inverse probability weights, where treated observations receive a weight of 1, and untreated observations receive a weight of $\frac{\hat{f}\left({\text{X}}_{\text{i}}\right)}{\text{1}-\;\hat{f}\left({\text{X}}_{\text{i}}\right)}$. Accordingly, the weights can be defined as:


$$w_{i}=\;D_{i}+\left(\text{1}-\;D_{i}\right)\;.\;\frac{\hat{f}\left({\text{X}}_{\text{i}}\right)}{\text{1}-\;\hat{f}\left({\text{X}}_{\text{i}}\right)}$$ (3)

Where $w_{i}$ is the weight assigned to observation i in the outcome regression model, $\hat{f}$ are the estimated propensity scores, ${\text{X}}_{\text{i}}$ is a vector of observed covariates for firm i. Next, Following Wooldridge [45], we estimate separate regression models for treated and untreated firms:


$$\begin{array}{c} {ATET}_{RA}=\;\frac{\text{1}}{n_{A}}\sum_{i=\text{1}}^{n}D_{i}\left[r_{A}\left(X_{i}\text{, }\delta_{A}\right)-\;r_{N}(X_{i}\text{, }\delta_{N})\right]\text{\textbackslash{} } \end{array}$$ (4)

In equation (4) we represent the average treatment effect on the treated (ATET) using the regression adjustment (RA) method. In this formulation, ${ATET}_{RA}$ is computed by averaging the difference between the predicted outcomes under treatment and under control, but only for those units that actually received the treatment. The term $n_{A}$ denotes the number of treated firms in the sample, The function $r_{A}\left(X_{i}\text{, }\delta_{A}\right)$ indicates the predicted outcome for firm i using a regression model estimated on the treated group, with $\delta_{A}\text{\textbackslash{} }$representing the corresponding parameter vector. Similarly, $r_{N}(X_{i}\text{, }\delta_{N})$ denotes the counterfactual outcome for the same firm predicted using a regression model fitted to the untreated group, with $\delta_{N}$ being the estimated parameters for that model.

Finally, the IPWRA estimator combines equations (3) and (4) to yield a doubly robust estimate of the treatment effect:


$$\begin{array}{c} {ATET}_{IPWRA}=\;\frac{\text{1}}{n_{A}}\sum_{i=\text{1}}^{n}D_{i}\left[r_{A}^{*}\left(X_{i}\text{, }\delta_{A}^{*}\right)-\;r_{N}^{*}(X_{i}\text{, }\delta_{N}^{*})\right]\text{\textbackslash{} } \end{array}$$ (5)

In this expression, ${ATET}_{IPWRA}$ is calculated by averaging the difference between the predicted outcome under treatment and the counterfactual prediction under control for firms that actually received the treatment. The term $r_{A}^{*}\left(X_{i}\text{, }\delta_{A}^{*}\right)$ represents the predicted outcome for firm i based on a regression model estimated using only the treated group, where the model is weighted by the inverse of the estimated propensity scores, and $\delta_{A}^{*}$ denotes the corresponding parameter vector. Similarly, $r_{N}^{*}(X_{i}\text{, }\delta_{N}^{*})$ is the predicted counterfactual outcome for the same firm using a model estimated on the untreated group, also weighted using the inverse probability weights, with $\delta_{N}^{*}$ being its associated parameters.

A critical assumption for the validity of the IPWRA estimator is the overlap (or common support) assumption. This requires that, for every combination of covariates $X_{i}$, there is a positive probability of both receiving and not receiving the treatment (i.e., the propensity score is strictly between 0 and 1). Meeting this assumption ensures that treated and untreated groups are comparable and that the model can properly estimate the counterfactual outcomes [49,50]. Failure to satisfy overlap can lead to biased estimates and increased variance, as the method cannot reliably infer treatment effects for regions of the covariate space with limited or no data for either group.

The data used in this study were collected by the Economic Research Forum (ERF), which conducted phone interviews in 2023 using a structured questionnaire. The ERF obtained the necessary approvals and complied with ethical guidelines for data collection. As the research utilized anonymized, secondary data collected by ERF, no additional ethical clearance was required by the authors. All data collection was carried out by ERF researchers based in Egypt. The data come from the transition to clean energy enterprise survey in Egypt, targeting businesses with fewer than 100 employees that began operations prior to 2023. Due to the absence of an official registry covering this population, the sampling frame was constructed using the Egypt Yellow Pages, which included over 288,000 businesses across all sectors and provinces. A systematic random sample of 20,623 businesses was selected from the Yellow Pages database. The sampling design was implicitly stratified by province and business sector to ensure broad sectoral and geographic coverage. While the sampling frame may not capture every eligible business, it is one of the most comprehensive business directories available in Egypt and includes firms across diverse economic activities.

Survey weights were applied to adjust for both selection probabilities and nonresponse. Design weights were calculated as the inverse of selection probabilities, and nonresponse adjustment factors were computed based on weighted response rates by region. The final weights were normalized to ensure that the weighted sample accurately represents the distribution of businesses in the original sampling frame. The overall response rate was 4.86%, with 1,002 completed surveys out of 20,623 sampled firms. Despite this relatively low response rate, common in phone-based business surveys, the stratified sampling design and use of nonresponse-adjusted weights support the generalizability of the findings. Moreover, key characteristics of the weighted sample align with known distributions of firm size and sectoral composition within Egypt, enhancing confidence in the external validity of the results. The unit of observation in the survey was the enterprise, and no personally identifiable or human-subject data were collected. As such, the study did not require institutional review board (IRB) approval. The questionnaire included two consent questions: one to obtain verbal consent to proceed with the interview, and another to obtain consent for audio recording. The survey did not involve minors, and all questions were strictly related to business operations rather than personal or sensitive information.

The survey’s central policy question was: “Are the majority of power outages in your area announced ahead of time?” Responses were coded as 1 for ‘mostly announced’ and 0 otherwise. Additionally, to control for the impact of outages, the survey collected additional data on blackout frequency, operational disruptions, and backup systems. Finally, the study controlled for firm-specific characteristics, such as firm age and size, as well as manager-specific characteristics, including gender and education level. These additional variables allow us to better isolate the effect of power outage announcements on business resilience and response. We show the detailed definitions in Table 1.

**Table 1:** Variables Definitions.

Revenue	Is a dummy variable = 1 if the revenues range from 5000000 to 50000 and 0 if less than 50000.
**Policy**	Are the majority of outages in your area announced ahead of time? 1 if mostly announced and 0 mostly unannounced.
**Blackouts**	Dummy variable = 1 if there are blackouts and 0 otherwise.
**Blackouts Frequency**	In a typical month, how many outages/blackouts of the grid happen? Daily Once/Twice a Week, Once/Twice a Month, never.
**Outage Effect**	Is a dummy variable = 1 wasted, damaged goods, not operating, not serving and 0 if no effect.
**Firm Size**	How many workers in total (paid/unpaid, or full time/part time) does this establishment have?
**Backup**	What is your main back-up source of electricity during outages from the grid? The variable is dummy variable = 1 if the backup is generator battery or storage device or solar lantern/lighting system and 0 otherwise.
**Manger Experience**	How many years of experience working in this field does the top manager have?
**CEO Gender**	Is the top manager male or female, dummy variable = 1 if male and 0 if female.
**Formality**	Does the establishment have a tax registration (formal registration)? A dummy variable = 1 if Yes and = 0 if No.
**Sector**	Agriculture or fishing or mining 1, textile and garments 2, industry of food 3, industry of mechanics or electronics or vehicles or services of motor vehicles 4, leather or chemical or petroleum or plastics or rubber or metallic products 5, wood products or furniture or paper and publishing 6, construction or utilities 7, retail 8, wholesale 9, transportation and storage 10, accommodation and food services 11, information and communication or IT 12, financial activities or real estate 13, education or health 14, other manufacturing or services 15.

### Descriptive statistics

A word about the data, before moving on to discuss the results: on average, about 69.8% of firms report positive revenue outcomes across the entire sample, with a slight variation between the two policy groups. Firms with access to advance outage announcements exhibit a marginally higher revenue success rate 71.1% compared to firms without announcements 68.3%. Only 22.5% of firms across the sample have backup power systems in place. This reflects the high cost and limited access to alternative power sources for many businesses. Firm size and managerial experience provide additional context about the resources available to these firms. Managerial experience is relatively high, with an average of 13.8 years, showing minor variation between the two groups. Table 2 below summarizes the data used.

**Table 2:** Summary statistics: All and by Policy.

	N	mean	sd	min	max
**Revenue**	995	0.698	0.459	0	1
**Blackouts frequency**	995	2.236	1.215	1	4
**Outage effect**	995	0.62	0.486	0	1
**Backup**	995	0.225	0.418	0	1
**Firm Size**	995	6.813	11.126	1	98
**Manger Experience**	962	13.816	11.436	0	60
**CEO Gender**	995	1.326	0.469	1	2
**Policy: 0**					
**Revenue**	448	0.683	0.466	0	1
**Blackouts frequency**	448	2.377	1.162	1	4
**Outage effect**	448	0.596	0.491	0	1
**Backup**	448	0.221	0.415	0	1
**Firm Size**	448	7.176	11.533	1	98
**Manger Experience**	432	13.722	11.777	0	60
**CEO Gender**	448	1.308	0.462	1	2
**Policy: 1**					
**Revenue**	547	0.711	0.454	0	1
**Blackouts frequency**	547	2.121	1.246	1	4
**Outage effect**	547	0.64	0.48	0	1
**Backup**	547	0.229	0.42	0	1
**Firm Size**	547	6.516	10.783	1	98
**Manger Experience**	530	13.892	11.16	0	55
**CEO Gender**	547	1.34	0.474	1	2

Table 3 reports Spearman’s rank correlation coefficients among the key explanatory variables. Overall, the correlations are low to moderate, indicating that multicollinearity is unlikely to pose a significant problem in the regression analysis. The most notable correlation is a moderately strong negative relationship between gender of manager and manager experience 0.43, suggesting that female managers tend to have more experience than their male counterparts.

**Table 3:** Spearman’s rank correlation coefficients.

Variables	(1)	(2)	(3)	(4)	(5)	(6)	(7)	(8)
**(1) Revenue**	1.000							
**(2) Blackouts frequency**	−0.110	1.000						
**(3) Outage Effect**	0.017	−0.066	1.000					
**(4) Backup**	−0.022	0.102	−0.121	1.000				
**(5) policy**	0.029	−0.110	0.041	−0.079	1.000			
**(6) Firm Size**	−0.204	0.009	0.131	−0.212	−0.013	1.000		
**(7) Manger Experience**	−0.044	0.212	0.070	0.073	0.029	0.209	1.000	
**(8) CEO Gender**	0.160	−0.210	−0.117	−0.120	0.039	−0.230	−0.436	1.000
**Spearman rho = −0.436**								

**Fig 1:**
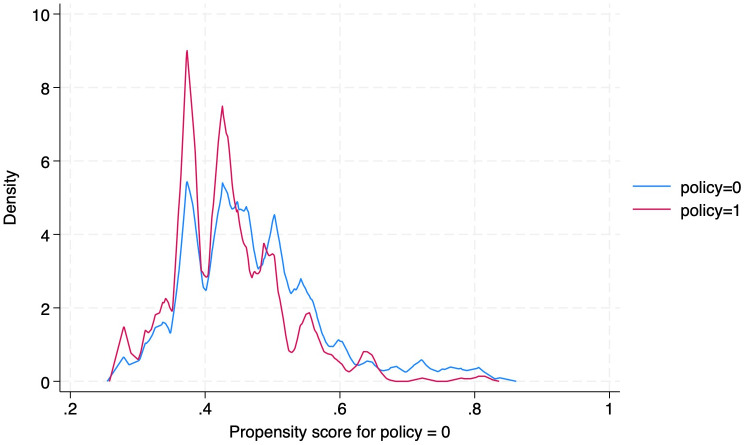
Propensity Matching Score Overlap Graph.

Furthermore, Fig 1 shows the overlap blue represents the companies that have found matching samples in the control group, and the red represents the samples of companies that have found suitable matching samples in the treatment group, thereby demonstrating the common support region essential for valid causal inference. In Table 4 we present the results of base regression models examining the relationship between electricity-related variables (such as blackout frequency and backup power) and the likelihood of positive revenue outcomes for firms. Most notably, blackout frequency is negative and statistically significant at the 1% level in both probit models, with values of −0.114 and −0.121. We find that when the frequency of blackouts increases, the probability of achieving a positive revenue outcome decrease. In other words, firms that experience more frequent power outages are less likely to have high revenue outcomes. Similarly, in the OLS models, blackout frequency has a negative and highly significant coefficient (−0.0381 and −0.0400). Thus, frequent blackouts are associated with a reduction in revenue, reinforcing the idea that electricity instability has a detrimental effect on firm performance.

**Table 4:** Base Regression, Probit and OLS.

	(1)	(2)	(3)	(3)	(4)	(4)
Variables	Probit	Probit	OLS	VIF	OLS	VIF
**Blackouts frequency**	−0.114***	−0.121***	−0.0381***	1.01	−0.0400***	1.09
	(0.0349)	(0.0374)	(0.0118)		(0.0124)	
**Outage effect**	0.123	0.132	0.0398	1.01	0.0435	1.03
	(0.0868)	(0.0912)	(0.0296)		(0.0302)	
**Backup**	0.551***	0.423***	0.169***	1.00	0.124***	1.07
	(0.111)	(0.119)	(0.0343)		(0.0358)	
**Firm Size**		0.0456***			0.00776***	1.10
		(0.00750)			(0.00141)	
**Manger Experience**		−0.00227			−0.000732	1.24
		(0.00424)			(0.00140)	
**CEO Gender**		0.127			0.0266	1.26
		(0.105)			(0.0343)	
**Constant**	0.597***	0.245	0.721***		0.653***	
	(0.108)	(0.218)	(0.0370)		(0.0709)	
**Observations**	995	962	995		962	
**R-squared**			0.038		0.069	

Standard errors in parentheses.

*** p < 0.01, ** p < 0.05, * p < 0.1.

Given the important role of securing energy resources, we find backup has a positive and highly significant in both Probit models, with coefficients of 0.551 and 0.423. The results imply that having a backup power system substantially increases the probability of achieving positive revenue outcomes. Therefore, firms with backup systems are better equipped to maintain operations during outages, which likely contributes to improved revenue performance. In Table 4 we can also see that firm size has a positive, suggesting that larger firms are more likely to achieve positive revenue outcomes. This may be because of larger firms having more resources to manage disruptions or greater operational flexibility to adapt to outages. Additionally, as shown in Table 4, the variance inflation factor (VIF) values for all explanatory variables remain well below the common threshold of 5, with the highest VIF observed for CEO gender at 1.26. Hence, the model satisfies a key assumption of linear regression and allows for reliable inference regarding the individual effects of each variable.

## Results and discussion

The results, presented in Tables 5 and 6, reveal the significant effect of blackouts, the mitigating role of advance notices, and the influence of firm-level and sectoral characteristics. These findings support and extend the literature on infrastructure reliability and firm performance in developing economies [15,16] while also offering new perspectives on the effectiveness of policy interventions. The policy of providing advance notice of power cuts emerges as a significant intervention to mitigate the adverse effects of outages. Firms under the announcement policy exhibit a 0.706 increase in revenue, compared to 0.69 for firms without such notices. The results of our model suggest advance announcements enable firms to better prepare for outages, potentially by rescheduling production, activating backup systems, or reallocating resources. However, the interaction effects in the treatment model estimates (TME1) reveal that the policy’s effectiveness is limited in high-blackout areas, where the coefficient for blackout frequency remains negative and significant. This suggests that while advance notices are beneficial, they are not a panacea for firms operating in regions with chronic electricity shortages. That is being said, we indeed need a complementary infrastructure improvement to address the root causes of electricity unreliability

**Table 5:** Treatment-effects estimation.

Revenue	Coef.	St.Err.	t-value	p-value	[95% Conf	Interval]	Sig
**POmeans**							
**Policy 0**	.69	.021	32.30	0.000	.648	.732	***
**Policy 1**	.706	.02	36.10	0.000	.668	.744	***
**OME0**							
**Blackouts frequency**	−.257	.096	−2.68	.007	−.444	−.069	***
**Outage Effect**	.297	.251	1.18	.238	−.196	.789	
**Backup**	.771	.34	2.27	.023	.105	1.438	**
**Firm Size**	.108	.069	1.57	.117	−.027	.243	
**Manger Experience**	.007	.011	0.69	.49	−.013	.028	
**CEO Gender**	.755	.284	2.66	.008	.199	1.311	***
**Textile & Garments**	−.641	.949	−0.68	.499	−2.501	1.219	
**Industry of Food**	−1.083	.867	−1.25	.212	−2.781	.616	
**Industry of Mechanic**	.107	.892	0.12	.905	−1.642	1.856	
**Leather/ Chemical**	−.562	.91	−0.62	.537	−2.345	1.221	
**Wood Products**	.201	1	0.20	.841	−1.758	2.16	
**Construction**	.383	1.133	0.34	.735	−1.837	2.604	
**Retail**	−.018	.815	−0.02	.982	−1.616	1.579	
**Wholesale**	.78	.971	0.80	.422	−1.124	2.684	
**Transportation**	7.441	1.033	7.21	0	5.417	9.465	***
**Accommodation**	.276	.905	0.30	.761	−1.498	2.05	
**Information**	.071	1.042	0.07	.945	−1.97	2.113	
**Financial Activities**	.158	1.017	0.16	.876	−1.836	2.152	
**Education or Health**	−.903	.905	−1.00	.318	−2.677	.87	
**Other Manufacturing**	−.972	.848	−1.15	.252	−2.635	.69	
**Constant**	−.366	1.033	−0.35	.723	−2.39	1.659	
**OME1**							
**Blackouts frequency**	−.165	.088	−1.88	.06	−.336	.007	*
**Outage Effect**	.251	.223	1.13	.26	−.186	.689	
**Backup**	.489	.292	1.68	.093	−.082	1.061	*
**Firm Size**	.138	.038	3.60	0	.063	.213	***
**Manger Experience**	−.01	.011	−0.93	.351	−.031	.011	
**CEO Gender**	.035	.252	0.14	.889	−.459	.529	
**Textile & Garments**	.725	1.269	0.57	.568	−1.763	3.212	
**Industry of Food**	1.313	1.518	0.86	.387	−1.663	4.288	
**Industry of Mechanic**	.667	1.274	0.52	.6	−1.83	3.165	
**Leather/ Chemical**	1.048	1.337	0.78	.433	−1.572	3.669	
**Wood Products**	1.302	1.385	0.94	.347	−1.413	4.017	
**Construction**	.747	1.4	0.53	.594	−1.998	3.491	
**Retail**	1.117	1.219	0.92	.36	−1.273	3.506	
**Wholesale**	1.16	1.276	0.91	.364	−1.342	3.661	
**Transportation**	7.394	1.378	5.37	0	4.694	10.094	***
**Accommodation**	3.443	1.566	2.20	.028	.373	6.513	**
**Information**	1.173	1.75	0.67	.503	−2.256	4.602	
**Financial Activities**	8.593	1.26	6.82	0	6.125	11.062	***
**Education or Health**	1.535	1.302	1.18	.239	−1.018	4.087	
**Other Manufacturing**	.329	1.26	0.26	.794	−2.141	2.798	
**Constant**	−.606	1.265	−0.48	.632	−3.085	1.873	
**TME1**							
**Blackouts frequency**	−.155	.055	−2.79	.005	−.264	−.046	***
**Outage Effect**	.227	.139	1.63	.103	−.046	.501	
**Backup**	.002	.167	0.01	.992	−.325	.329	
**Textile & Garments**	.533	.515	1.04	.3	−.476	1.541	
**Industry of Food**	.446	.563	0.79	.428	−.657	1.549	
**Industry of Mechanic**	.942	.53	1.78	.076	−.098	1.981	*
**Leather/ Chemical**	1.062	.543	1.95	.051	−.003	2.127	*
**Wood Products**	.179	.599	0.30	.765	−.995	1.353	
**Construction**	.343	.638	0.54	.592	−.908	1.593	
**Retail**	.781	.465	1.68	.093	−.13	1.693	*
**Wholesale**	1.213	.523	2.32	.02	.188	2.239	**
**Transportation**	−.095	.949	−0.10	.92	−1.954	1.764	
**Accommodation**	.923	.532	1.74	.083	−.119	1.966	*
**Information**	−.691	.73	−0.95	.344	−2.122	.741	
**Financial Activities**	.63	.679	0.93	.354	−.701	1.96	
**Education or Health**	1.041	.537	1.94	.052	−.011	2.093	*
**Other Manufacturing**	.732	.499	1.47	.142	−.245	1.71	
**Constant**	−.337	.488	−0.69	.49	−1.293	.62	
**Mean dependent var**	**0.695**			**SD dependent var**	**0.460**		

*** p < .01, ** p < .05, * p < .1.

**Table 6:** Treatment-effects estimation (Added Formality).

Revenue	Coef.	St.Err.	t-value	p-value	[95% Conf	Interval]	Sig
**Policy 0**	.688	.022	31.98	0	.646	.73	***
**Policy 1**	.706	.02	36.06	0	.668	.745	***
**OME0**							
**Blackouts frequency**	−.263	.097	−2.71	.007	−.453	−.073	***
**Outage Effect**	.248	.251	0.99	.323	−.244	.741	
**Backup**	.731	.346	2.11	.035	.053	1.41	**
**Firm Size**	.097	.065	1.49	.135	−.03	.225	
**Manger Experience**	.006	.01	0.55	.583	−.015	.026	
**CEO Gender**	.858	.292	2.94	.003	.285	1.43	***
**Formality**	−.598	.266	−2.25	.024	−1.12	−.077	**
**Textile & Garments**	−.652	1.012	−0.64	.519	−2.636	1.331	
**Industry of Food**	−1.175	.916	−1.28	.2	−2.969	.62	
**Industry of Mechanic**	.05	.959	0.05	.959	−1.83	1.93	
**Leather/ Chemical**	−.543	.98	−0.55	.58	−2.464	1.378	
**Wood Products**	.257	1.074	0.24	.811	−1.849	2.362	
**Construction**	.255	1.146	0.22	.824	−1.992	2.502	
**Retail**	−.085	.891	−0.10	.924	−1.831	1.661	
**Wholesale**	.644	1.04	0.62	.536	−1.394	2.682	
**Transportation**	7.231	1.042	6.94	0	5.189	9.273	***
**Accommodation**	.285	.978	0.29	.771	−1.632	2.202	
**Information**	−.053	1.078	−0.05	.961	−2.166	2.06	
**Financial Activities**	−.019	1.084	−0.02	.986	−2.144	2.107	
**Education or Health**	−1.004	.98	−1.02	.306	−2.926	.917	
**Other Manufacturing**	−1.037	.92	−1.13	.26	−2.84	.767	
**Constant**	.466	1.191	0.39	.696	−1.869	2.801	
**OME1**							
**Blackouts frequency**	−.173	.088	−1.96	.05	−.347	0	**
**Outage Effect**	.239	.228	1.05	.295	−.208	.685	
**Backup**	.49	.29	1.69	.091	−.078	1.059	*
**Firm Size**	.129	.036	3.56	0	.058	.2	***
**Manger Experience**	−.012	.011	−1.15	.251	−.034	.009	
**CEO Gender**	.091	.253	0.36	.718	−.405	.587	
**Formality**	−.371	.227	−1.63	.102	−.816	.074	
**Textile & Garments**	.662	1.307	0.51	.613	−1.901	3.225	
**Industry of Food**	1.193	1.546	0.77	.44	−1.837	4.223	
**Industry of Mechanic**	.582	1.315	0.44	.658	−1.995	3.158	
**Leather/ Chemical**	.941	1.379	0.68	.495	−1.762	3.643	
**Wood Products**	1.247	1.427	0.87	.382	−1.551	4.045	
**Construction**	.691	1.442	0.48	.632	−2.135	3.517	
**Retail**	1.036	1.258	0.82	.41	−1.43	3.503	
**Wholesale**	1.053	1.318	0.80	.425	−1.531	3.636	
**Transportation**	7.524	1.429	5.26	0	4.722	10.325	***
**Accommodation**	3.395	1.596	2.13	.033	.266	6.523	**
**Information**	.937	1.778	0.53	.598	−2.548	4.422	
**Financial Activities**	8.349	1.301	6.42	0	5.8	10.898	***
**Education or Health**	1.404	1.343	1.05	.296	−1.227	4.035	
**Other Manufacturing**	.244	1.3	0.19	.851	−2.303	2.79	
**Constant**	0	1.359	−0.00	1	−2.663	2.663	
**TME1**							
**Blackouts frequency**	−.147	.056	−2.62	.009	−.256	−.037	***
**Outage Effect**	.25	.141	1.78	.075	−.025	.526	*
**Blackouts frequency**	.021	.167	0.12	.901	−.306	.348	
**Formality**	.211	.149	1.42	.156	−.081	.502	
**Textile & Garments**	.498	.513	0.97	.331	−.506	1.503	
**Industry of Food**	.437	.559	0.78	.434	−.658	1.533	
**Industry of Mechanic**	.948	.527	1.80	.072	−.085	1.981	*
**Leather/ Chemical**	1.065	.542	1.96	.049	.002	2.127	**
**Wood Products**	.169	.597	0.28	.778	−1.001	1.338	
**Construction**	.35	.632	0.55	.579	−.888	1.589	
**Retail**	.76	.461	1.65	.099	−.144	1.664	*
**Wholesale**	1.227	.519	2.37	.018	.211	2.244	**
**Transportation**	−.11	.937	−0.12	.906	−1.948	1.727	
**Accommodation**	.933	.53	1.76	.078	−.105	1.971	*
**Information**	−.655	.73	−0.90	.369	−2.085	.775	
**Financial Activities**	.666	.675	0.99	.324	−.657	1.99	
**Education or Health**	1.067	.534	2.00	.046	.021	2.113	**
**Other Manufacturing**	.727	.495	1.47	.143	−.245	1.698	
**Constant**	−.641	.53	−1.21	.227	−1.679	.398	

*** p < .01, ** p < .05, * p < .1.

The frequency of blackouts has a consistently negative and statistically significant impact on firm revenue across all models. In the no-announcement condition, a one-unit increase in blackout frequency reduces revenue by 25.7%. The findings suggest a severe operational disruption caused by unpredictable power outages. This finding is consistent with the previous literature, which emphasizes how unreliable electricity supply increases production costs, disrupts workflows, and reduces output [51]. Interestingly, when firms receive advance notice of outages, the negative impact of blackout frequency diminishes slightly but remains significant, with a coefficient of −0.165. This result suggests that while advance announcements provide some relief, they are insufficient to fully counteract the challenges posed by frequent outages.

Additionally, we find that the backup systems significantly enhance firm resilience to outages. In the no-announcement condition, firms with backup systems experience a 77.1% increase in revenue, point out the importance of proactive investments in resilience-building measures. This aligns with the findings of Rud [52], who argues that firms with backup infrastructure are better equipped to withstand infrastructure shocks. Similarly, firm size plays a protective role, with larger firms demonstrating a 13.8% increase in revenue under the announcement policy. This suggests that larger firms, with their greater resources and adaptive capacities, are better positioned to leverage advance notices and implement contingency plans [20]. Notably, there is remarkable sectoral differences in how firms respond to outages and advance announcements. The transportation sector stands out as particularly resilient. This resilience may stem from the sector’s lower dependency on continuous power or its ability to adapt operations to intermittent supply. Similarly, the financial activities sector shows remarkable gains under the announcement policy. This suggests that advance notices are especially beneficial for sectors that rely heavily on uninterrupted power for client services and data security. In contrast, the accommodation sector benefits significantly from advance announcements. This is likely due to the high costs associated with unplanned outages in this industry, where customer satisfaction and operational continuity are critical. The wholesale sector also shows a positive interaction with the policy, indicating that advance notices help firms manage inventory and logistics more effectively during planned outages. The analysis also highlights the role of CEO gender in shaping firm outcomes. In the no announcement condition, firms led by male CEOs experience a 75.5% increase in revenue compared to those led by female CEOs. This finding raises important questions about gender disparities in access to resources, networks, or decision-making authority, which may influence firm resilience to outages.

Including formality as a variable in Table 6 reveals important insights into how regulatory compliance affects firm outcomes. In the absence of prior outage announcements, formal firms those registered with tax authorities or complying with labor and regulatory requirements, experience a 59.8% reduction in revenue compared to informal firms. This substantial decline suggests that formal enterprises may bear higher fixed and compliance-related costs (e.g., taxes, labor standards, and licensing fees) that make them less flexible and more vulnerable to sudden electricity disruptions. In contrast, informal firms, which typically operate outside the regulatory framework, may be better positioned to adapt to unannounced outages due to lower overhead costs and more informal, adaptable operational structures. Nevertheless, while this result highlights the potential cost of formality under unreliable infrastructure, it should be interpreted with caution. As McKenzie and Sakho [53] emphasize, the productivity advantages or disadvantages of formality depend heavily on local institutional quality, enforcement practices, and access to public services. In contexts where infrastructure is unreliable and enforcement is uneven; formality may impose costs without delivering commensurate benefits. Therefore, the negative association between formality and revenue in this setting likely reflects a structural disadvantage that formal firms face in adjusting to electricity shocks without prior warning.

**Fig 2:**
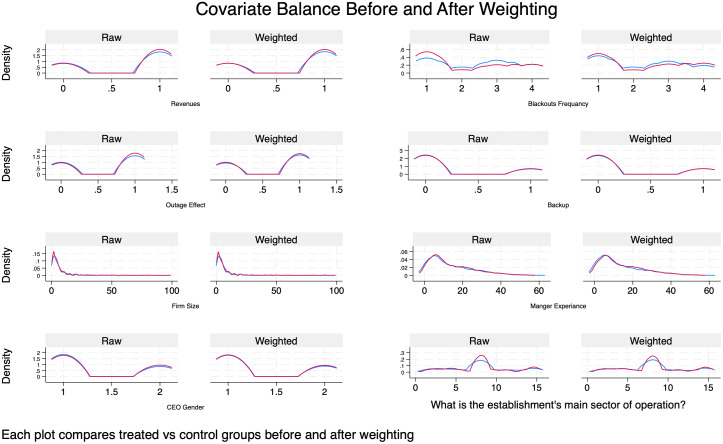
Covariate balance after and before weighting for each variable.

**Fig 3:**
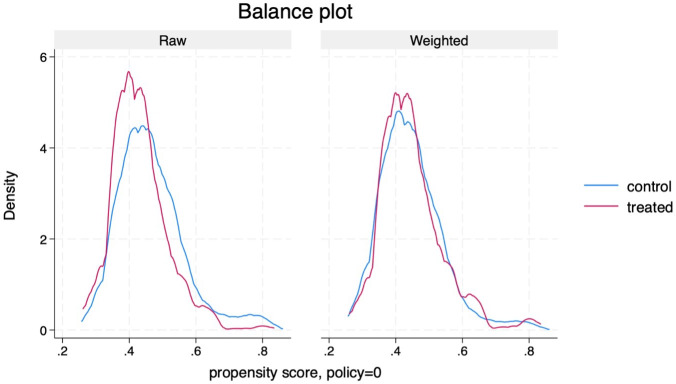
Covariate balance after and before weighting.

To assess the validity of our causal estimates, it is essential to assess whether the treatment and control groups are comparable based on their observed characteristics. Specifically, we examine whether covariates are balanced across groups before and after weighting, which is a key condition for satisfying the conditional independence assumption (CIA) as shown in Table 7 and Figs 2 and 3. In Table 7, the “Raw” column, show that several covariates such as blackouts frequency and sector categories have standardized mean differences that indicate imbalance, meaning the treated and untreated firms differ systematically on these variables. However, after applying inverse probability weighting using regression adjustment (IPWRA), the “Weighted” column shows that these differences are greatly reduced and very close to zero. This suggests that the weighting process has effectively balanced the covariates across groups, which helps reduce selection bias. In econometric terms, this improves the plausibility of the conditional independence assumption (CIA), which states that potential outcomes are independent of treatment assignment given observed covariates. Moreover, the variance ratios also approach 1 after weighting, indicating similar dispersion of covariates across groups as a further sign of good balance.

**Table 7:** Covariate balance summary.

	Raw	Weighted		
**Number of obs** =	940	940.0		
**Treated obs** =	520	469.9		
**Control obs =**	420	470.1		
	**Standardized**	**differences**	**Variance**	**Ratio**
	**Raw**	**Weighted**	**Raw**	**Weighted**
**Blackouts frequency**	−0.204	0.000	1.150	1.200
**Outage Effect**	0.089	−0.002	0.953	1.001
**Blackouts frequency**	0.083	−0.002	0.955	1.001
**Backup**	0.015	−0.004	1.021	0.995
**Textile & Garments**	−0.051	0.000	0.844	1.000
**Industry of Food**	−0.065	0.001	0.732	1.005
**Industry of Mechanic**	0.042	−0.006	1.165	0.978
**Leather/ Chemical**	0.068	0.000	1.325	1.002
**Wood Products**	−0.100	−0.001	0.565	0.997
**Construction**	−0.075	0.002	0.613	1.012
**Retail**	0.040	0.003	1.016	1.001
**Wholesale**	0.124	−0.004	1.526	0.986
**Accommodation**	0.042	0.003	1.180	1.011
**Information**	−0.181	0.005	0.237	1.035
**Financial Activities**	0.050	−0.003	1.215	0.987
**Education or Health**	−0.008	0.002	0.979	1.005

Additionally, Fig 3 show the distribution of propensity scores between the treated and control groups before and after applying inverse probability weighting and present that the weighting process has effectively balanced the distribution of propensity scores across treatment groups. As a result, the IPWRA framework is more likely to yield unbiased estimates of treatment effects. Additionally, the overidentification test in Table 8 confirms this: the high p-value (0.9719) means we do not reject the null hypothesis that covariates are balanced, suggesting that our propensity score model is well specified. Overall, these diagnostics provide confidence that the model adequately addresses observable confounding, allowing us to interpret the estimated treatment effects as causal under the assumption of no hidden bias.

**Table 8:** Overidentification test.

chi2(19) =	9.09583
**Prob>chi2 =**	0.9719

Overall, the results highlight hat advance notification of electricity outages is associated with improved revenue outcomes for SMEs. This suggests that predictable power supply, even when constrained, can support better planning and reduce economic losses. In fact, these findings echo earlier studies on firm resilience under infrastructure constraints. As firms with predictable disruptions experience less severe productivity losses compared to those with sudden and frequent blackouts [5,16]. For example, Fisher-Vanden et al. [16] argue that firm responses to energy shocks depend heavily on their ability to anticipate and adapt to these shocks. Yet, the policy’s effectiveness is uneven. Firms located in high-blackout areas derive fewer benefits from announcements, suggesting that predictability alone may not be enough in contexts of extreme power unreliability. Complementary investments in energy infrastructure are critical for maximizing the impact of policy tools. Backup systems, such as generators, also appear to play a key role, particularly among larger firms with more resources. Additionally, firms in transportation, storage, and accommodation services are more responsive, likely due to their operational sensitivity to power supply. Another contribution of this study is its emphasis on the role of formality. The interaction between formal status and announcement policy reveals that formal firms may benefit less from announcements under certain conditions, due to higher compliance costs or regulatory burdens. As Egypt continues to struggle with energy challenges, these insights offer valuable lessons for policymakers and stakeholders aiming to foster sustainable economic development.

While this study is the first of its kind that has devoted considerable efforts to understand how announced and transparent public policy may impact the SME performance in Egypt, it presents several methodological considerations that warrant further exploration. The use of Yellow Pages listings for data collection provided a comprehensive source for conducting phone surveys, future research could benefit from incorporating additional data sources to enhance sample representativeness, particularly of enterprises operating in the informal sector. The reliance on self-reported data, while common in firm-level studies, introduces potential response biases that could be mitigated in future research through triangulation with objective measures or administrative data. The generalizability of findings to other developing economies, while not the primary focus of this Egypt-specific study, opens avenues for comparative research across diverse economic and energy landscapes. Future research also may encompass longitudinal analyses to elucidate the temporal dynamics of outage announcement effects on SME productivity. Additionally, future research should also explore the long-term dynamics of firm adaptation to electricity disruptions and the role of renewable energy solutions in enhancing resilience. Furthermore, future research could investigate synergies between outage announcements and alternative energy solutions adopted by SMEs. Researchers should conduct comprehensive economic impact assessments to quantify how improved SME productivity from effective outage management policies affects the broader economy. Finally, further research is needed to disentangle the mechanisms through which formality influences firm resilience to electricity outages.

## Conclusions

This paper provides clear empirical evidence that electricity outages pose a substantial constraint on SME performance in Egypt. Notably, we show that advance announcements of outages, while not solving the underlying energy deficit, enable firms to organize production schedules, allocate resources more efficiently, and ultimately mitigate revenue losses. This finding is economically significant and offers an actionable policy lever in settings where service reliability cannot be guaranteed in the short term. Yet, the effectiveness of the policy is conditional. It varies across firm size, sector, and, critically, local blackout intensity. Firms operating in high-frequency outage zones, for example, derive limited benefit from announcements, as the scope for mitigation narrows with increasing disruption. Moreover, firms in sectors with time-sensitive production cycles (e.g., food services, transport) appear more sensitive to predictability. These heterogeneities matter and suggest that one-size-fits-all interventions will likely fall short.

However, several caveats are in order. First, our findings should be read in the context of Egypt’s broader energy transition. Investments in grid resilience, diversification of energy sources, and targeted support for backup systems are likely to yield more durable benefits than communication alone. Nevertheless, predictability is cheap, and its marginal return, as shown here, is far from negligible. Second, the observed variation in resilience by firm formality hints at deeper institutional frictions. Formal firms, often subject to regulatory compliance costs, may lack the flexibility enjoyed by informal enterprises. Whether this reflects policy failure or structural trade-offs requires further investigation, ideally using administrative or tax data that can track firm behavior over time. Finally, the policy implications extend beyond electricity. The principle of predictive governance, providing timely, credible information about future disruptions, has broader relevance in an increasingly uncertain business environment. Whether the shock is energy, inflation, climate-related, or geopolitical, firms benefit from early signals that allow for adaptive planning. In sum, outage announcements represent a modest but effective tool in the policymaker’s arsenal. But they are no substitute for the long-run investments in energy infrastructure, institutional reform, and SME finance that are ultimately necessary for productivity growth. Future research should deepen the understanding of firm-level adaptation, explore the complementarities between predictability and investment incentives, and examine how such policies play out across different governance and infrastructure contexts.
